# Sentinel Node Detection in Head and Neck Malignancies: Innovations in Radioguided Surgery

**DOI:** 10.1155/2009/681746

**Published:** 2009-11-09

**Authors:** L. Vermeeren, W. M. C. Klop, M. W. M. van den Brekel, A. J. M. Balm, O. E. Nieweg, R. A. Valdés Olmos

**Affiliations:** ^1^ Department of Nuclear Medicine, Netherlands Cancer Institute—Antoni van Leeuwenhoek Hospital, Plesmanlaan 121, 1066 CX Amsterdam, The Netherlands; ^2^ Department of Head and Neck Surgery and Oncology, Netherlands Cancer Institute—Antoni van Leeuwenhoek Hospital, Plesmanlaan 121, 1066 CX Amsterdam, The Netherlands; ^3^ Department of Surgery, Netherlands Cancer Institute—Antoni van Leeuwenhoek Hospital, Plesmanlaan 121, 1066 CX Amsterdam, The Netherlands

## Abstract

Sentinel node mapping is becoming a routine procedure for staging of various malignancies, because it can determine lymph node status more precisely. Due to anatomical problems, localizing sentinel nodes in the head and neck region on the basis of conventional images can be difficult. New diagnostic tools can provide better visualization of sentinel nodes. In an attempt to keep up with possible scientific progress, this article reviews new and innovative tools for sentinel node localization in this specific area. The overview comprises a short introduction of the sentinel node procedure as well as indications in the head and neck region. Then the results of SPECT/CT for sentinel node detection are described. Finally, a portable gamma camera to enable intraoperative real-time imaging with improved sentinel node detection is described.

## 1. Introduction

Sentinel lymph node biopsy is increasingly being used as a staging procedure for various malignancies. The sentinel node can be defined as the lymph node on the direct drainage pathway from the primary tumor [1]. Therefore, this particular node is likely to be the first node to harbor metastasis and can be used to provide information about the rest of the nodal basin. Based on the hypothesis of sequential tumor spread, sentinel node mapping can be used for nodal staging, being more precise than imaging procedures and less invasive than regional prophylactic nodal dissection. In melanoma, the sentinel node status has proven to provide relevant prognostic information and is widely performed to accurately stage melanoma patients [2]. The procedure has evolved to a routine staging method for patients with clinically localized breast cancer and is nowadays used to stage patients with other solid tumors as well. The role of sentinel node biopsy has not been clearly defined for head and neck squamous cell carcinoma. Positive sentinel nodes have shown to be a negative prognostic factor in oral cancer [3] and several authors have published good results regarding staging accuracy of the sentinel node biopsy in oral cancer patients [4–6]. It can be used to select patients for subsequent neck dissection and can reduce morbidity in many sentinel node negative patients who can be spared this operation.

Conventional planar lymphoscintigraphy is routinely used for preoperative sentinel node detection and localization. Dynamic planar images can show the draining lymph vessels directly after injection, while sequential static planar images show an overview of the number and localization of the sentinel nodes. Sentinel nodes are often clearly visualized with planar images and the levels of these nodes can be localized using external radioactive markers, such as a cobalt-57-source pen. Interpretation of planar images can be difficult because the anatomy information is limited to outlining the body contour. Especially sentinel nodes in the head and neck region can be difficult to localize, as a result of complex anatomy, interlacing lymphatic vessels, unexpected drainage patterns and because the three-dimensional surface of the structures of the head is not visualized on planar images. Furthermore, sentinel nodes in proximity to the injection area, for example, in the preauricular or submandibular region, can easily be missed on planar images.

Intraoperative sentinel node detection with the gamma ray detection probe can be challenging as well. More than 95% of the administered radioactivity stays behind at the injection site and may cause nearby located sentinel nodes to be missed. Distinguishing sentinel nodes from second-echelon nodes with the probe is not possible and, as no overview can be provided, certainty about removal of all radioactive sentinel nodes is not provided by the gamma probe. 

The complexity of the anatomy in the head and neck region requires optimization of sentinel node detection and localization in this area. First, we will report on the clinical indication for sentinel node mapping in head and neck malignancies. Then we will outline recent innovations to improve radioguided lymph node surgery in the head and neck area and we will report on our own preliminary results with those innovative imaging techniques.

## 2. Indication for Sentinel Node Mapping in the Head and Neck Region

As sentinel node mapping in the head and neck region is used for lymph node staging, only patients with clinically and radiologically negative lymph node assessment (stage N0) are considered for this procedure. If enlarged lymph nodes are found by physical examination or ultrasound aspiration cytology, the presence of nodal metastases can be confirmed and patients can proceed to selective or (modified) radical neck dissection. 

Controversy exists regarding the appropriate indication for sentinel node biopsy in melanoma and squamous cell carcinoma. The indication for sentinel node mapping in melanoma generally depends on the Breslow thickness of the tumor, although ulceration and other prognostic factors might also be taken into account [7]. In thin lesions, the low risk of finding nodal metastases can be a reason to omit sentinel node staging and many authors do not use sentinel node biopsy in such lesions (e.g., less than 0.75 mm or less than 1.0 mm) [8]. In thick lesions, the high risk of synchronous distant metastases may outweigh the possible therapeutic and prognostic benefits of lymphadenectomy or sentinel node mapping [9, 10]. 

The exact role of lymph node staging with sentinel node biopsy in oral cancer has not been clearly defined yet, though several authors use the procedure to stage T1 or T1 and T2 lesions [4–6]. A diagnostic meta-analysis by Paleri et al. showed a good sensitivity (92.6%) of the sentinel node procedure in squamous cell cancer of the oral cavity an oral pharynx [5], while Civantos Jr. et al. found a negative predictive value of 96% for this procedure [4]. The lesser morbidity of sentinel node biopsy is often used as an argument against elective neck procedures; however some authors find selective neck dissection more appropriate in view of the high risk of nodal metastasis [11]. This especially is true in patients with more advanced lesions.

## 3. SPECT/CT for Preoperative Sentinel Node Detection

**Figure 1:**
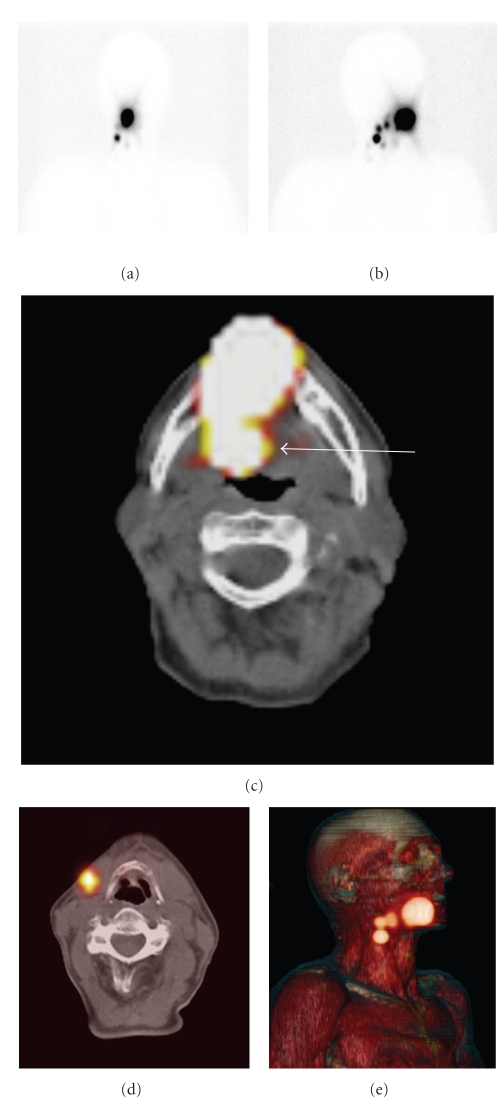
SPECT/CT to rule out a presumed sentinel node. Anterior (a) and oblique (b) planar static images after 2 hours show drainage to the right neck on the basis of which 3 sentinel nodes were marked. SPECT/CT (c) demonstrates the cranial hotspot located at the base of the tongue in the oropharynx (arrow), due to leakage of the tracer from the injection area. The sentinel nodes are clearly visualized with SPECT/CT (d), while three-dimensional reconstruction (e) shows an anatomic overview of all hotspots.

**Figure 2:**
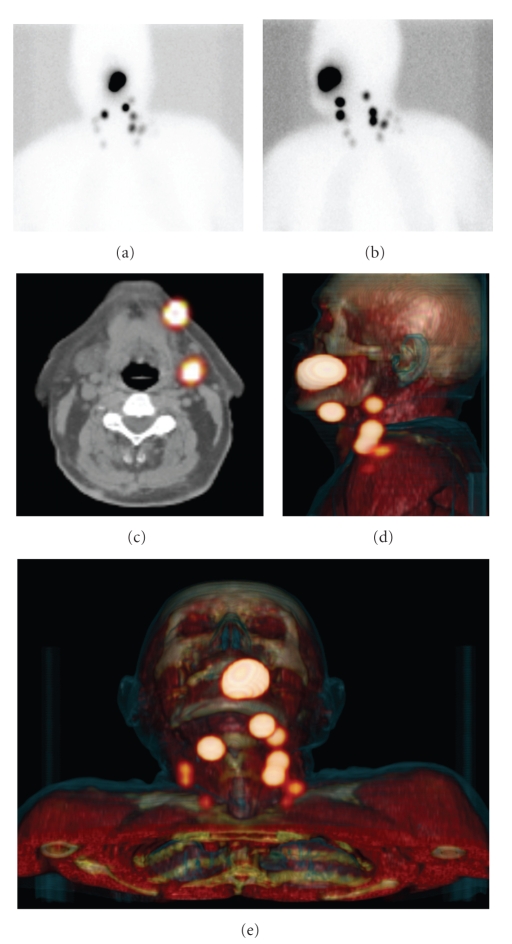
SPECT/CT localizing sentinel nodes and providing anatomic overview. Anterior (a) and oblique (b) planar static images after 2 hours show several hotspots. Two-dimensional SPECT/CT reconstruction exactly localizes each node, for example, localizing 2 sentinel nodes in the submandibular region (c). Three-dimensional SPECT/CT reconstruction shows an anatomic overview of all sentinel nodes (d) and (e).

Hybrid single-photon emission computed tomography with integrated computed tomography (SPECT/CT) is a multimodal imaging device and can be used to visualize and localize sentinel nodes [12–20]. SPECT/CT can optimize sentinel node visualization which may lead to improved intraoperative detection [21]. A SPECT/CT system generally consists of a dual-head variable-angle gamma camera equipped with low-energy high-resolution collimators and a multislice spiral CT optimized for rapid rotation. A matrix 128 × 128 is used for SPECT acquisition and 25 seconds per view using 4–6-degree-angle steps enable adequate images. The CT settings are aimed at obtaining a low-dose CT (e.g., 130 KV, 40 mAs, B30s kernel), which is appropriate for attenuation correction and mapping. A major advantage of the hybrid SPECT/CT system is that the patient need not be moved between the SPECT and the CT data acquisition. After reconstruction, the SPECT images are corrected for attenuation and scatter. Axial 5 mm SPECT and CT slices are usually generated. Subsequently, the SPECT and CT images are fused and can be displayed using multiplanar reconstruction projection with two-dimensional orthogonal reslicing in axial, sagittal, and coronal orientation and maximum intensity projection. A three-dimensional image display can also be constructed, based on volume rendering of fused SPECT/CT images with a 16-bit color look-up table with defined opacity for soft tissue, bone, and skin. Based on this three-dimensional view, the opacity can be manually adjusted to visualize these structures with different colors: red for muscle, ochre for bone, and blue for skin. The sentinel node is displayed in yellow (Figures 1 and 2) in both two-dimensional and three-dimensional display. Sentinel lymph nodes can be identified and localized on the two-dimensional images, while three-dimensional reconstruction gives an anatomic overview of all lymph nodes in relation to the injection area.

Several authors have reported on the use of SPECT/CT in head and neck malignancies; previous study details are summarized in Table 1. The injected dose varies among the different studies as well as planar imaging protocols and timing of SPECT/CT and sentinel node excision. The injected dose depends on the time to operation. A larger dose of radioactivity is needed with a longer interval because of the physical half-life of the radionuclide. A dosage of 10 MBq–60 MBq radioactive colloid is used when patients are operated on the same day, while 50 MBq–120 MBq is used when operation takes place the next day. If low dosages are used, lymphatic vessels may not be visualized, which leads to difficulty in identifying sentinel nodes.

The results of additional imaging with SPECT/CT are summarized in Table 2. Numbers of patients studied are rather small in all studies and exact drainage visualization numbers are not mentioned in every study. Recorded visualization of lymphatic drainage on planar images ranges from 83% to 100%. SPECT/CT has proven to visualize additional sentinel nodes in more than half of the studies [12, 13, 15, 17–19], although the authors of one study conclude that SPECT/CT rarely reveals sentinel nodes that are not detected on planar images [19]. Especially nodes adjacent to the injection area appear to be detected by SPECT/CT, while these are easily missed on planar images [13, 15]. In 3 out of 9 studies, presumed sentinel nodes could be interpreted as nonnodal tracer uptake (tracer leakage or contamination) on the basis of SPECT/CT images [12, 18, 19]. All authors agree that SPECT/CT provides useful localization information [12–20]. Covarelli et al. have proven the clinical relevance of this localization information, since the operation time was significantly less when sentinel node surgery was performed on the basis of SPECT/CT images compared to planar images [20].

Our own preliminary results with SPECT/CT in head and neck malignancies are in line with the literature findings. The visualization rate was 100% for planar imaging as well as SPECT/CT in the first 33 patients, but SPECT/CT visualized additional sentinel nodes in 18% of the patients and excluded hotspots as being sentinel nodes in 9% of all patients. Figure 1 shows an example of a hotspot that was assumed to be a sentinel node but which SPECT/CT showed to be caused by tracer leakage in the oral cavity on SPECT/CT. The exact anatomic localization could be realized with SPECT/CT in all patients. An example of anatomic localization with SPECT/CT is given in Figure 2. In our hospital, surgical incisions are based on SPECT/CT images, because of the anatomic localization and overview SPECT/CT images provide.

Combining our results with literature findings, SPECT/CT appears to be very useful for exact anatomic localization of the sentinel nodes. In the head and neck area it is of considerable importance to identify the relation of sentinel nodes to several vital vascular and neural structures in order to be able to safely remove these nodes. SPECT/CT also detects sentinel nodes that are missed on planar lymphoscintigraphy in a substantial number of patients. In head and neck cancer, many sentinel nodes are located in close proximity to the injection area and are easily overlooked on planar images. Cases of nonnodal tracer uptake (e.g., tracer leakage in the oral cavity after injection or contamination on the skin) can be identified with SPECT/CT, while distinguishing between leakage and a sentinel node on planar images is often impossible. 

After tracer administration (intradermally in melanoma, submucosally in oral cavity carcinoma), sequential planar images can identify nodes that are on a direct drainage pathway from the primary tumor (sentinel nodes). Subsequently, SPECT/CT can localize these sentinel nodes, giving anatomical reference points for the planning of the surgical approach.

## 4. Portable Gamma Cameras for Intraoperative Imaging

**Figure 3:**
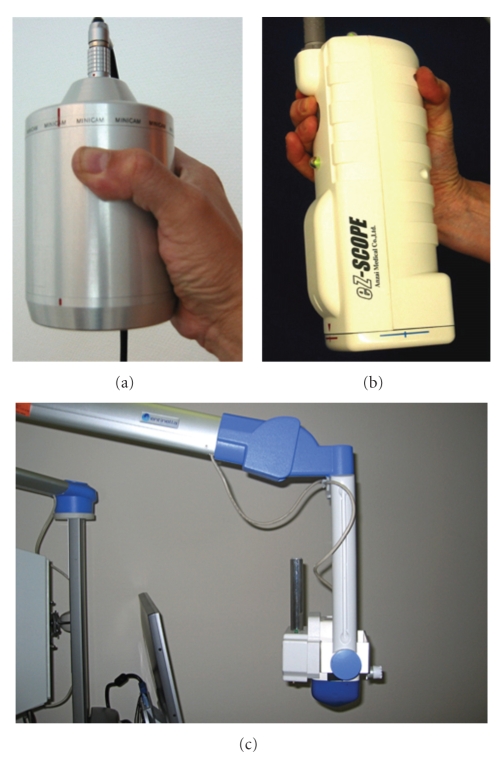
Development of portable gamma cameras. (a) First generation portable gamma camera with a weight of approximately 2 kg. (b) Portable gamma camera with a weight <1 kg but without support system. (c) Last generation portable gamma camera with improved ergometrical details and adequate support system for intraoperative use.

Sentinel node surgery is based on the combination of gamma probe detection and blue dye mapping. Surgeons localize sentinel nodes combining the auditive signal (probe) to the visual one (blue dye). In head and neck patients, the use of blue dye can be problematic. The injection of blue dye in the mouth can lead to obscured tumor edges and thus interfere with resection [18]. Furthermore, blue dye shifts very fast. For these reasons the application of blue dye in head and neck patients, with a high density of lymph nodes and short distances between injection site and sentinel node, is limited. The incorporation of another visual element in sentinel node surgery of the head and neck can facilitate the procedure. Against this background, portable gamma cameras have been designed to facilitate radioguided surgery. The development of such cameras is illustrated in Figure 3. While the first prototypes were heavy hand-held devices, the new generation of portable gamma cameras is equipped with a stable support system.

The use of portable gamma cameras has been described in parathyroidectomy [22, 23]. The parathyroid adenomas can be located intraoperatively by means of sestamibi imaging using a portable gamma camera [22, 23]. Another possible application of the portable gamma camera can be localizing lymph nodes [24–26]. Exact localization of sentinel nodes by the portable gamma camera was shown in an animal model [27], and recently the synchronous use of a portable gamma camera and gamma probe showed exact localization of sentinel nodes in 11 breast cancer patients [28]. Furthermore, the depth of the sentinel nodes was successfully estimated preoperatively using the portable gamma camera [28]. Several conditions are required to optimize intraoperative imaging with the portable gamma camera. A summary of these requirements is given in Table 3.

In our centre, the use of a portable gamma camera was introduced for laparoscopic lymph node localization in urological malignancies [29] and has recently been commenced to aid lymph node localization in oral cavity carcinoma as well. The portable gamma camera (Sentinella, S102, GEM imaging, Valencia, Spain) is equipped with a 4 mm pinhole collimator and uses a CsI(Na) continuous scintillating crystal. The pinhole collimator enables visualization of the whole surgical field and the field of view depends on the distance between the camera and the imaging plane. The field of view is 4 × 4 cm when placed at 3 cm from the imaging plane and increases to 20 × 20 cm when placed at a distance of 15 cm. The intrinsic spatial resolution is 1.8 mm. The extrinsic spatial resolution values are 7 mm and 21 mm for a distance of 3 cm and 15 cm, respectively. The detection sensitivity for this collimator depends on the distance to the imaging plane, being 708 and 41 cpm/uCi for distances of 3 cm and 15 cm respectively. These and other technical details of this portable gamma camera are described by Sánchez et al. [24]. 

**Figure 4:**
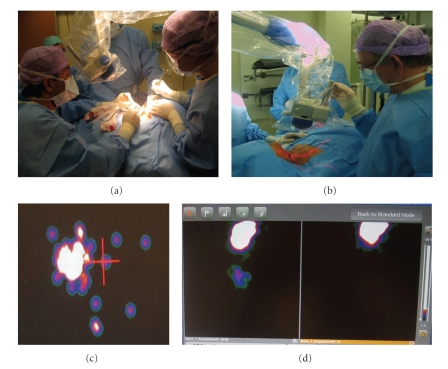
Localization and postexcision monitoring. Continuous monitoring (a) provides the possibility to record the whole procedure. With stepwise monitoring (b), the sentinel nodes are localized first, then excision takes place, and afterwards the portable gamma camera is used to screen for remaining activity. The laser pointer is positioned above the previous marked sentinel node level and the camera displays the technetium-signal (c), indicating that the node is located just right from the laser pointer. The portable gamma camera can also give an overview of the surgical field (d). It shows the injection area with a sentinel node located more caudally. After excision, the camera clearly shows no remaining radioactivity (d).

Intraoperative real-time imaging with the portable gamma camera provides an overview of all radioactive hotspots in the whole surgical field. Its position can be adjusted to also show sentinel nodes near to the injection area, which are easily overlooked using the probe. The differentiation between sentinel nodes and secondary tier is facilitated, because the amount of radioactivity within each node can be quantified with the portable gamma camera and the intraoperative images can be related to the preoperative scintigraphic images. Furtheremore, sentinel nodes can be exactly localized by on screen visualization, besides the audiological localization by the laparoscopic gamma probe. Figure 4 shows 2 possibilities for intra-operative use of the portable gamma camera. Continuous monitoring can be used to record the whole procedure and stepwise monitoring enables localization of sentinel nodes and detects remaining activity afterwards. If the laser pointer signal matches the technetium signal on screen, this indicates that the sentinel node has been exactly localized. Another clear advantage of the portable gamma camera is the certainty it can provide about the completeness and accuracy of the sentinel node excision, since it shows remaining radioactivity. Figure 4 also shows the comparison of the situation before and after excision. 

As experience with radioguided surgery is gained, the need for advanced imaging modalities will increase. The portable gamma camera is an innovative tool that can improve nodal excision in areas with complex anatomy. Further research in our institute is underway to define the exact value and indication of the intraoperative use of a portable gamma camera.

## 5. Future Directions

Over the next years, the use of SPECT/CT might become routine procedure for patients with difficult to interpret planar images. The major challenge remains to optimize intra-operative visualization of sentinel nodes. The development of a gamma camera with a multiplanar detection system (e.g., two heads) might enable real-time three-dimensional visualization. Furthermore, the development of new tracers may improve intraoperative visualization as well. A slower migrating alternative to patent blue might improve the direct intraoperative localization of sentinel nodes in the neck. Another option may be the development of a dual-tracer, which contains radioactivity as well as color and can be used for lymphoscintigraphy and intraoperative visual and auditive (gamma probe) at the same time.

## 6. Conclusion

Sentinel node biopsy is increasingly being used to provide accurate staging in early stage head and neck malignancies, such as melanoma and oral squamous cell carcinoma. Lymphatic mapping in the head and neck area can be complicated because of the complex anatomy and variable drainage patterns in this area and easy obscuration of sentinel node by the primary injection site. The need for accurate imaging extends beyond planar lymphoscintigraphy. 

SPECT/CT is a new imaging modality that improves preoperative lymphatic mapping by exactly localizing sentinel node. It depicts sentinel nodes that are missed on planar images, especially nodes in close proximity to the injection area, and can exclude presumed sentinel nodes that are based on tracer leakage or contamination. Sequential planar images remain essential to distinguish sentinel nodes from second-echelon nodes.

A portable gamma camera can be used to improve intraoperative search for sentinel nodes. Such a camera provides real-time imaging of the sentinel node and can detect and localize those nodes, even if located near the injection area. A gamma camera increases certainty about the accuracy and completeness of the excision of the radioactive nodes, since it facilitates postexcision monitoring.

## Figures and Tables

**Table 1:** Technical details of SPECT/CT in head and neck malignancies in various studies.

Study	N*	Malignancy	Dose	Injection	Planar Imaging	Timing SPECT/CT	SPECT	Surgery	Details
Even-Sapir et al. [12]	9	3 HNM 6 OCC	74 MBq	Intradermal or submucosal injection 4 peritumoral deposits of 0.4 mL each	Sequential images within minutes after injection until visualization (up to 24 hours)	Not specified	3° angle/20 s to 25 s steps Matrix: 128 × 128	Next day	Total number of patients: 34 (9 head and neck malignancy)
									
Wagner et al. [13]	30	OCC	20 MBq	Intra-mucodermal injection 2 peritumoral deposits of 0.05 mL each	Static image 60 minutes after injection	60 minutes after injection	6^˚^ angle/30 s steps Matrix: 128 × 128	Same day	Sentinel node biopsy performed in 13/30
									
Lopez et al. [14]	10	OCC	22.2 MBq	4 peritumoral deposits Total volume <0.5 mL	Sequential images 4 to 24 hours after injection	Not specified	6^˚^ angle/10 s steps Matrix: 128 × 128	Same day	Image registration performed manually by reprojection
									
Thomsen et al. [15]	37	OCC	20 MBq	4–6 peritumoral submucosal deposits Total volume 0.2 mL	Static images 30–60 minutes after injection	Not specified	6^˚^ angle/8 s steps Matrix: 128 × 128	Same day	SPECT/CT in 37 out of 40 patients
Terada et al. [16]	12	OCC	18.5 MBq	4 peritumoral submucosal deposits, volume unclear	A static lymphoscintigram (anterior and bilateral oblique) was performed	After planar images	Not specified	Same day	Results of SPECT/CT are not compared to results of planar imaging
									
Bilde et al. [17]	34	OCC	120 MBq or 60 MBq^§^	4 peritumoral submucosal deposits Total volume 0.2 mL	Dynamic imaging (lateral and anterior) during 20 minutes Static images after 30 and 90 minutes	After planar images	3^˚^ angle/30 s steps Matrix: 128 × 128	Some the next day, some the same day	
									
Khafif et al. [18]	20	OCC	74 MBq	Injection at the border of the primary tumor 4 deposits of 0.4 mL each	Sequential images within minutes until visualization (up to 24 hours)	Not specified	3^˚^ angle / 20 s–25 s steps Matrix: 128 × 128	Next day	
Keski-Säntti et al. [19]	15	OCC	74 MBq	Peritumoral injection in 1 or 2 deposits Total volume 0.2 mL	Planar lymphoscintigraphy with anterior and lateral projections	Not specified	Not specified	Next day	
Covarelli et al. [20]	12 versus 11**^±^**	HNM	50 MBq or 10 MBq^§^	Peritumoral intradermal injection in 4 deposits In case of excision: 2 deposits around surgical scar Total volume 0.1 mL	Dynamic planar imaging for 20 minutes Sequential static images up to until 3 hours	45 minutes after injection	4^˚^ angle / 30 s steps Matrix: 256 × 256	Some the next day, some the same day	Patients received either planar imaging or SPECT/CT

*number of patients with a head and neck malignancy that received SPECT/CT.

HNM: head and neck melanoma.

OCC: oral cavity carcinoma.

^§^ the first dose was injected if patients were operated the next day; the last dose was injected if patients were operated the same day.

^±^ 12 patients received SPECT/CT only; 11 patients received planar imaging only.

**Table 2:** SPECT/CT results in various studies.

Study	Visualization with planar imaging	Visualization with SPECT/CT	Additional sentinel nodes visualized with SPECT/CT	Main conclusions with regards to imaging
Even-Sapir et al. [12]	Multiple drainage basins: 11%	Multiple drainage basins: 33% Additional clinical relevant information with SPECT/CT: 44%	In 3 out of 9 patients 1 false positive node excluded	SPECT/CT provides additional data of clinical relevance in patients with trunk or head and neck melanoma and patients with mucosal head and neck tumor.
Wagner et al. [13]	38 sentinel nodes	Sentinel node visualization with planar imaging and SPECT/CT: 90% 49 sentinel nodes	11 sentinel nodes	SPECT/CT is feasible for sentinel node detection. SPECT/CT enhances topographic orientation and diagnostic sensitivity. SPECT/CT is necessary to identify nodes adjacent to the primary lesion.
Lopez et al. [14]	Sentinel node visualization: 100%	Localization of the sentinel nodes in 9/10 patients		Multimodal registration is an effective method for anatomic localization of the sentinel nodes in N0 oral squamous cell carcinoma.
Thomsen et al. [15]	99 sentinel nodes	SPECT/CT and added oblique planar images: 123 sentinel nodes	24 extra sentinel nodes found with SPECT/CT in combination with added oblique planar images	Added oblique planar images and/or SPECT/CT detect extra clinical relevant hotspots in 38% of the patients. Sentinel lymph nodes close to injection area are difficult to find.
Terada et al. [16]		Sentinel node visualization with planar imaging and SPECT/CT: 100%		Intraoperative sentinel node biopsy based on SPECT/CT images is an easy, accurate, and reliable method. Analysing the three hottest sentinel nodes reliably predicts a patients neck status.
Bilde et al. [17]	88 sentinel nodes	Sentinel node visualization: 94% 107 sentinel nodes	19 sentinel nodes In 15 out of 32 patients (47%)	Correction of anatomic level with SPECT/CT in 22%. Reclassification of anatomic level during surgery in 22%. SPECT/CT detects more sentinel nodes and provides additional anatomical and spatial information.
Khafif et al. [18]		Sentinel node visualization with planar imaging and SPECT/CT: 95% SPECT/CT added significant anatomical preoperative information in 6 out of 20 patients (30%)	Additional sentinel nodes seen in 2 patients (metastatic sentinel node in 1) Exclusion of sentinel nodes in 4 patients (all activity at injection site)	SPECT/CT sentinel node mapping provides additional preoperative data of clinical relevance.
				
Keski-Säntti et al. [19]	Sentinel node visualization: 100%	Sentinel node visualization: 100% Additional data provided by SPECT/CT was considered clinical relevant in 6 out of 15 patients (40%)	1 additional sentinel node visualized 2 false positive nodes excluded	SPECT/CT enables more accurate localization of sentinel nodes. SPECT/CT rarely reveals sentinel nodes that are not detected on planar images.
				
Covarelli et al. [20]	Sentinel node visualization: 83% 12 sentinel nodes in 12 patients	Sentinel node visualization: 100% 13 sentinel nodes in 12 patients		SPECT/CT is more effective and reliable than planar lymphoscintigraphy. Sentinel node biopsy takes significantly less time in the SPECT/CT group.

**Table 3:** Requirements for intra-operative imaging.

Portable gamma camera	(1) Manageable design (portable and stable)
	(2) Sufficient resolution and detection sensitivity
	(3) No delay between image acquisition and display (real-time imaging)
	(4) Adequate field of view

Intra-operative situation	(5) No interference with field of operation
	(6) Possibility for continuous monitoring
	(7) Spatial orientation on screen
	(8) Possibility to use pointers for position and localization
	(9) Real-time quantification of the number of counts per second

Sentinel node	(10) Sufficient uptake of the radiotracer by the sentinel node
